# Identification of two robust subclasses of sepsis with both prognostic and therapeutic values based on machine learning analysis

**DOI:** 10.3389/fimmu.2022.1040286

**Published:** 2022-11-25

**Authors:** Wei Zhou, Chunyu Zhang, Zhongwei Zhuang, Jing Zhang, Chunlong Zhong

**Affiliations:** ^1^ Department of Anesthesiology, Huzhou Central Hospital, The Affiliated Huzhou Hospital, Zhejiang University School of Medicine, Huzhou, Zhejiang, China; ^2^ Department of Neurosurgery, Shanghai East Hospital, School of Medicine, Tongji University, Shanghai, China; ^3^ Department of Neurosurgery, Shanghai East Hospital, Nanjing Medical University, Nanjing, China; ^4^ Institute for Advanced Study, Tongji University, Shanghai, China

**Keywords:** sepsis, clustering, LASSO, logistic regression, clinical outcomes

## Abstract

**Background:**

Sepsis is a heterogeneous syndrome with high morbidity and mortality. Optimal and effective classifications are in urgent need and to be developed.

**Methods and results:**

A total of 1,936 patients (sepsis samples, n=1,692; normal samples, n=244) in 7 discovery datasets were included to conduct weighted gene co-expression network analysis (WGCNA) to filter out candidate genes related to sepsis. Then, two subtypes of sepsis were classified in the training sepsis set (n=1,692), the Adaptive and Inflammatory, using K-means clustering analysis on 90 sepsis-related features. We validated these subtypes using 617 samples in 5 independent datasets and the merged 5 sets. Cibersort method revealed the Adaptive subtype was related to high infiltration levels of T cells and natural killer (NK) cells and a better clinical outcome. Immune features were validated by single-cell RNA sequencing (scRNA-seq) analysis. The Inflammatory subtype was associated with high infiltration of macrophages and a disadvantageous prognosis. Based on functional analysis, upregulation of the Toll-like receptor signaling pathway was obtained in Inflammatory subtype and NK cell-mediated cytotoxicity and T cell receptor signaling pathway were upregulated in Adaptive group. To quantify the cluster findings, a scoring system, called, risk score, was established using four datasets (n=980) in the discovery cohorts based on least absolute shrinkage and selection operator (LASSO) and logistic regression and validated in external sets (n=760). Multivariate logistic regression analysis revealed the risk score was an independent predictor of outcomes of sepsis patients (OR [odds ratio], 2.752, 95% confidence interval [CI], 2.234-3.389, P<0.001), when adjusted by age and gender. In addition, the validation sets confirmed the performance (OR, 1.638, 95% CI, 1.309-2.048, P<0.001). Finally, nomograms demonstrated great discriminatory potential than that of risk score, age and gender (training set: AUC=0.682, 95% CI, 0.643-0.719; validation set: AUC=0.624, 95% CI, 0.576-0.664). Decision curve analysis (DCA) demonstrated that the nomograms were clinically useful and had better discriminative performance to recognize patients at high risk than the age, gender and risk score, respectively.

**Conclusions:**

In-depth analysis of a comprehensive landscape of the transcriptome characteristics of sepsis might contribute to personalized treatments and prediction of clinical outcomes.

## Introduction

Sepsis, a comprehensive syndrome with great heterogeneity, is related to disappointingly high mortality and morbidity, caused by dysregulated host systemic inflammatory and immune response to infection (1, 2). The insights into the host immune response have advanced remarkably, however, previous research contributes a little to the mainstays of prevention, early recognition and supportive care, and the development of novel therapeutic strategies (3). The main obstacles to improvement are the absence of a precise and accurate definition of the disorder, which includes a large number of multi-dimensional clinical and biological characteristics. Comprehensive analysis of features might contribute to the discovery of undescribed subsets or phenotypes, which help to evaluate the risk of clinical outcomes and the response to clinical interventions (4). For example, Scicluna et al. reported a classification system, using machine learning analyses on blood genomic data of sepsis samples, which posted Mars1 subset of sepsis was remarkably related to mortality (5). Bhavani et al. developed septic sub-phenotypes based on large-scale clinical analysis and revealed that the confirmed four sub-phenotypes could have different landscapes of inflammation markers and clinical outcomes (6). However, these features failed to illustrate critical pathophysiological changes and demonstrated underlying mechanisms and processes.

A high percentage of studies have displayed the genome-wide expression profiling of sepsis. The availability of a large number of genome-wide expression profiling from public databases, such as Gene Expression Omnibus (GEO) and ArrayExpress, supplies great opportunities to discover and identify accurate and effective prognostic and predictive signatures. The unsupervised analysis allows the researchers to classify and define disease subgroups on genome-wide expression data (7, 8). Meanwhile, recent advances in meta-clustering and data pooling have substantially improved the unrobust performance caused by subtle changes in the clustering methods, or small datasets (9). The data-driven analysis has successfully defined and validated clinically relevant disease subgroups in several diseases (10, 11). In addition, clustering analysis on whole blood gene expression confirmed the higher mortality subgroup characterized by immune exhaustion and the other sub-phenotype with a lower death rate has the upregulation of proinflammatory processes (12).

The present research comprehensively analyzed publicly available transcriptomic profiles of sepsis cohorts. A panel of sepsis-associated candidate features were identified to classify the septic samples into two subgroups. According to the functionality and activity of molecules and differences in immune cell composites, the cluster was named as Adaptive subgroup and cluster B was named as Inflammatory subcluster. In addition, samples in Adaptive subgroup demonstrated a lower mortality rate than the other. Then, a risk factor was established, which might be promised in sepsis to predict prognosis and guide clinical personalized management.

## Methods and materials

### Data acquisition and processing

**Figure 1:**
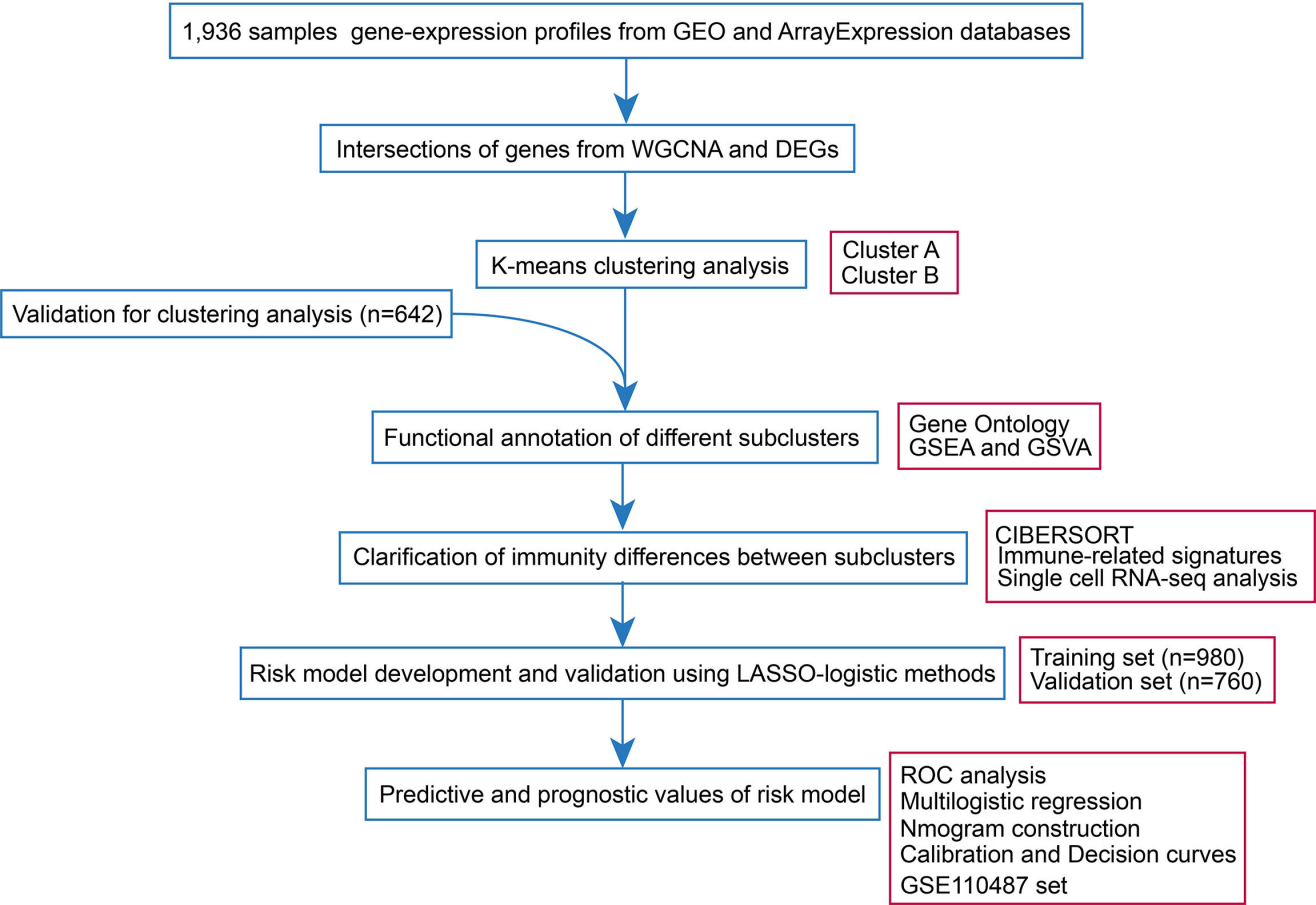
Workflow of the present research.

Sepsis datasets were downloaded from the GEO database and ArrayExpress database. Probes were annotated by the corresponding documents and Probes with missing gene symbols were excluded. Ensemble ID was annotated into gene symbol by R package ‘org.Hs.eg.db’. The mean value of expression was adopted, when there were multiple probe sets mapping to the same gene symbol. The missing value of expression datain the sepsis samples were dealt with R package ‘impute’. Samples were excluded if they included absent follow-up information such as age and gender. In addition, patients with age<18 were also excluded. Characteristics of included samples were demonstrated in **Table S1**. It should be noted, that age is the strong risk factor associated with sepsis owing to the fact that those over 65 years of age have a more than 10-fold higher incidence rate of the disease compared with those between 18 and 49 years (13). In addition, it has been estimated that in half of the aged, functional impairments occur rather than complete recovery (14). Therefore, we grouped the patients into ≥65 and <65 years for further analysis. The raw count data of RNA-sequencing (RNA-seq) were transformed into transcripts per million (TPM)-quantified data. The batch effect was removed by combat function in the ‘sva’ R package. The workflow of the study was shown in **Figure 1**.

### Weighted gene co-expression network analysis

WGCNA was introduced here to explore potential genes related to sepsis biology. Genes with top 80% highest variance in 1,936 samples were selected. The scale-free network was constructed when the soft threshold was β=18, Subsequently, the adjacency matrix was transformed into a topological overlap matrix (TOM). The correlation of modules with sample traits could be calculated to figure out sepsis-related modules. Genes with module membership (MM)>|0.80| and gene significance (GS)>|0.20| were included for further analysis.

### K-means clustering analysis

K-means analyses were conducted based on the training and the combined five validation sets. no age and gender differences between the two cohorts, tested by Chi-square test (**Table S2**). Differentially expressed genes (DEGs) between normal samples and sepsis samples were analyzed by ‘limma’ R package and genes with log2FC>|1.0| and FDR<0.05 were considered significant. After intersecting DEGs and candidate genes from WGCNA, the remained key candidate genes were subjected to K-means clustering analysis. The number of clusters was determined by the elbow method (EM) and average silhouette method (ASM). And the principal component analysis (PCA) plot was used to display the clustered samples. DEGs with log2FC>|1.0| and FDR<|0.05| between different clusters were also identified and visualized with volcano plot.

### Inference of immune infiltrates and single cell RNA-seq analysis

Aberrant immune reprogramming exerts significant effects on sepsis pathobiology (15). For quantification of immune infiltrates in sepsis samples, the Cibersort algorithm was introduced on the training cohort, with 1,000 permutations preset. Immune infiltrates of sepsis cohorts were divided into two groups, separately, in accordance with the clusters from k-means analysis. Immune cell markers were obtained from previous research (16). We got a matrix of those immunocytes and visualized this result *via* an R package ‘ggplot2’. Accessible scRNA-seq data acquisition (GSE151263) was downloaded from the GEO database, and four sepsis samples were subject for in-depth research (17). The scRNA-seq data was processed with the R package ‘Seurat’ (18). Specific cell markers were obtained for cell category annotation from the CellMarker database (19).

### Development of signature related to sepsis survival

980 sepsis samples with complete survival information in 5 datasets including GSE54514, E-MTAB-4451, GSE65682, GSE185263 and GE131761 in the discovery set were treated as a training set to calculate the risk score. And 760 objects from E-MTAB-4421, E-MTAB-5273, E-MTAB-7581, and GSE95233 cohorts were included as a validation set. There were no age and gender differences between the two groups (training and validation sets), tested by Chi-square test (**Table S3**). The 90 candidate features obtained by genes from WGCNA and DEGs were subjected to the least absolute shrinkage and selection operator (LASSO) regularization (α=1) using the glmnet package. The survival-related features were identified using a 10-fold stratified cross-validation to differentiate between non-survivor and survivor controls in the training set. Risk score was then computed for each sepsis sample using the logistic regression model in the training set. We applied a method to calculate the risk score for each sample with sepsis, the formula was as follows: $ris\text{k}\;score=log\{0.941*\sum_{1}^{n}(coefi\times expi)\}$ where coef was the coefficient calculated from logistic regression analysis. Multivariable logistic regression models adjusted by gender and (<65 and ≥65 years) age group were used to identify the independently predicting performance of risk score in differentiating survivor from non-survivor sepsis individuals. Nomograms were constructed including age, gender and risk score, and decision curve analysis (DCA) was used to quantify net benefits at different threshold probabilities. The receiver operating characteristic (ROC) curves and 95% confidence intervals (CI) were generated for assessment of model performance.

### Enrichment analysis

Gene ontology (GO) and gene set enrichment analysis (GSEA) analyses were conducted on DEGs by ‘clusterProfiler’ R package (20) and the enrichment terms were considered significant with a strict cutoff false discovery rate (FDR) of less than 0.05. Meanwhile, gene set variation analysis (GSVA) (21) was performed to estimate variations of pathway activity over a sample population in an unsupervised manner, with ‘h.all.v7.5.1.entrez.gmt’ as a reference set. To explore the correlation between the sepsis signature and other relevant biological processes, 14 gene sets were curated including CD8 T-effector signature; antigen processing machinery; immune-checkpoint; pan-fibroblast TGFβ response signature (Pan-F-TBRS); DNA replication-dependent histones and etc. (22). The markers of the corresponding biological processes were deposited in **Table S4**.

### Statistical analysis

For comparisons of two groups, statistical significance for normally distributed variables was estimated by student t-test. The categorical variables were analyzed on the root of chi-square test. Correlation coefficients were computed by Pearson correlation analyses. To identify significant genes in the differential gene analysis, Bonferroni-Hochberg (B-H) method was introduced to calculate false discovery rate (FDR). Heatmap was visualized by the R package ‘pheatmap’. R package ‘forestplot’ was employed to display the findings of survival analysis of candidate genes in training dataset. The predicting accuracy of the established risk signature, area under the curve (AUC) and 95% confidence interval (CI) were computed based on the ‘pROC’ package. All statistical analyses were conducted using R (v4.1.0) and SPSS software (version 25.0). Two-sided P<0.05 were considered statistically significant.

## Results

### Weighted gene co-expression network analysis and gene selection

**Figure 2:**
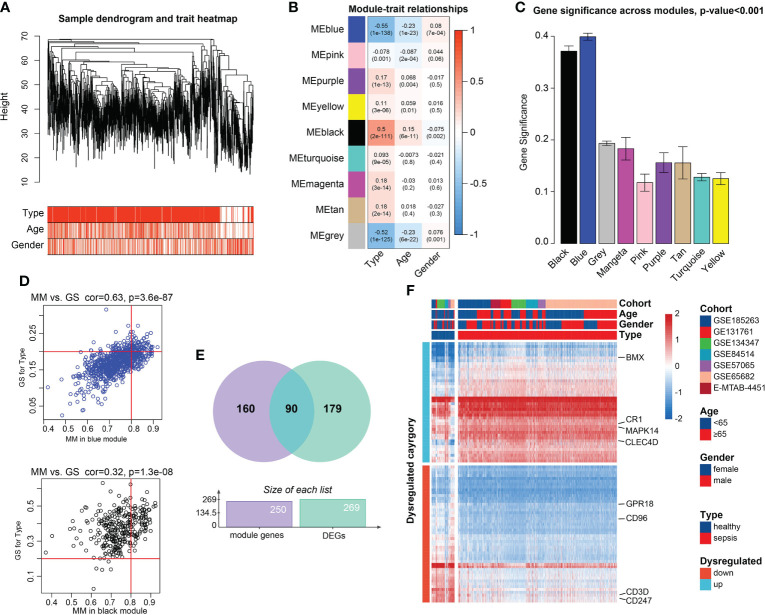
Candidate genes detection. **(A)** Clustering dendrogram of the saved 1,778 sepsis samples in WGCNA and clinical features. **(B)** Heatmap of Pearson correlation analysis of modules and clinical traits. Rows represent modules and columns represent traits. The values ​​in the squares represent correlation degree and p values. Color red represents positive correlation and color blue represents negative correlation. **(C)** Boxplots of GS among 9 modules. Module blue and module black demonstrated higher values gene significance, than that of the 7 modules, tested by t-test. **(D)** Scatter plots of Correlation of GS within MM. Genes with |GS |>0.2 and |MM |>0.8 were considered significant. **(E)** Venn plot of the intersections between DEGs and genes filtered from WGCNA. **(F)** Heatmap of the candidate genes. The expression values were normalized from -2 to 2. Color red represents relatively increased expression and color blue represents relatively deceased expression.

PCA demonstrated the 1,936 samples (sepsis samples, n=1,692; normal samples, n=244) in 7 datasets had tremendous batch effect, including GSE54514, GSE57065, GSE65682, GSE131761, E-MTAB-4451, GSE185263, and GSE134347 (**Figure S1A**). And by ‘sva’ R package, we could observe that the batch effect was significantly removed (**Figure S1B**). Genes with top 80% highest variance, that is, 5,052 genes were selected to carry out WGCNA. Then sample clustering was conducted to detect outliers, with average parameters in hclust function. 1,778 sepsis samples were left for subsequent analysis, which were displayed in **Figures S2A** and **2A**. With soft-threshold power value set as 18 (**Figure S2B**), the corresponding R^2^ reached up to 0.99, meeting the standard of scale-free topology (**Figure S2C**). Modules with similarity>0.8 were combined, and 9 modules were saved out of 13 modules (**Figure S2D**). Module-trait correlation degree was calculated, in which the blue and black modules demonstrated great correlation with type trait (blue module: cor=-0.55, p=1e-138; black module: cor=0.50, p=2e-111, **Figure 2B**). The average gene significance in each module was computed, and modules black and blue had the higher the mean gene significance values than those in the other 7 modules (**Figure 2C**). Finally, 250 genes with |GS|>0.20 and |MM|>0.80 were filtered out in the both modules (**Figure 2D**). After intersection of module genes and DEGs between normal and sepsis samples, 90 DEGs associated with sepsis were filtered out (**Figure 2E**). Among them, CD3D, CD247, CD96 and G Protein-Coupled Receptor 18 (GPR18) were found to be relatively overexpressed in normal blood samples and BMX Non-Receptor Tyrosine Kinase (BMX), Mitogen-Activated Protein Kinase 14 (MAPK14), Complement C3b/C4b Receptor 1 (CR1), and C-Type Lectin Domain Family 4 Member D (CLEC4D) were found to be upregulated in sepsis samples (**Figure 2F**).

### K-means clustering analysis

**Figure 3:**
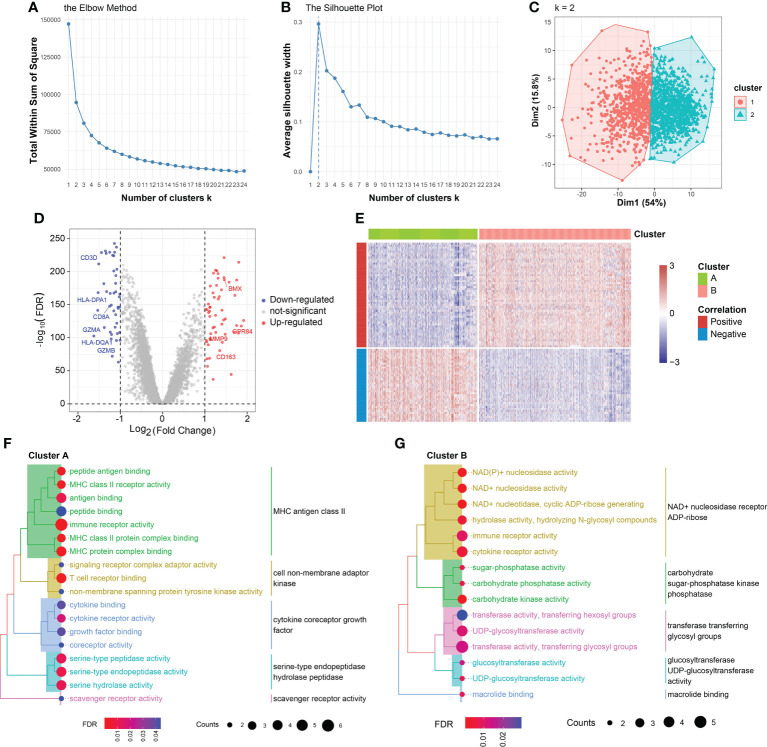
K-means clustering analysis and cluster annotation. **(A)** Total within sum of square (WSS) plotted against the number of clusters. The WSS dropped rapidly from 1 to 2 classes and slowly after k = 2. **(B)** Average silhouette width plotted against the number of clusters, demonstrating the 2-subclass was the ideal choice. **(C)** Scatter plot of distribution of sepsis samples in the two principal dimensions. **(D)** Volcano plot of DEGs of cluster B *vs*. cluster A. **(E)** Heatmap of DEGs between cluster A and cluster B. The expression values were normalized from -3 to 3. Color red represents relatively increased expression and color blue represents relatively deceased expression. **(F)** Gene Ontology (GO) analysis on DEGs overexpressed in cluster A. **(G)** Gene Ontology (GO) analysis on DEGs overexpressed in cluster B.

In the training set, 1,692 sepsis samples were selected for K-means cluster analysis. A total of representative 90 genes were obtained in the combined 7 cohorts. Clustering analysis was performed on the 90 candidate features. The optimal numbers of clusters were determined to be two by measuring the total within sum of square and average silhouette width (**Figures 3A, B**). The two classes could be well separated in the first two major dimensions (**Figure 3C**). There were 703 patients in cluster A (41.5%) and 989 patients in cluster B (58.5%). In the training set, there were 980 samples had complete survival information, and then the association between prognosis and cluster findings was calculated. Patients divided into class B demonstrated remarkably disadvantageous clinical outcomes, in comparison with that in cluster A (60.7% [145/239] *vs*. 39.3% [94/364]; p=0.044, Chi-square test) (**Table S5**). Meanwhile, there was a significant increase of number of sepsis patients with age≥65, when compared with patients in cluster B (59.9% [276/461] *vs*. 40.1% [185/461]; p=0.004, Chi-square test) (**Table S5**). However, no difference of gender distribution was observed between the two subgroups (Female: 47.1% [193/410] *vs*. 52.9% [217/410]; Male: 43.7% [249/570] *vs*. 56.3% [321/570], p=0.299, Chi-square test) (**Table S5**). As shown in **Figure S3**, the heatmap demonstrated that distinct molecule features between cluster A and cluster B. Particularly, T cell-related markers such as CD3D and CD3E were relatively overexpressed in the cluster A subgroup. CLEC4D, critical in mediating the infiltration of myeloid cells, was comparatively upregulated in cluster B (**Figure S3**). External validation is a key component of any exercise in clustering. Therefore, we carried out the K-means clustering analysis on 617 samples in 5 independent datasets and the combined five cohorts from GEO and ArrayExpress databases. The clear batch effect among the 5 cohorts were corrected by ‘sva’ package **Figures S4A, B**. Clustering analysis on each new dataset produced 2 robust clusters, as shown in **Figure S5A**.

### Differential expression analysis

Differential expression analysis in the training set revealed 99 genes were expression-dysregulated between the two classes (FDR<0.05 and log2|FC|>1.0, **Figure 3D**). In addition, there were 56 DEGs overexpressed in cluster A and 43 DEGs expression-upregulated in cluster B. Among them, we found CD3D, CD3G, and CD3E displayed increased expression levels in cluster A and Interleukin 18 Receptor Accessory Protein (IL18RAP), Interleukin 1 Receptor Associated Kinase 3 (IRAK3), and BMX demonstrated elevated expression levels in cluster B (**Figure 3D**). The correlation of DEGs with sample subtypes were calculated by Pearson correlation analysis. As shown in **Figure 3E**, the correlation degrees>0 were defined positive and degrees<0 were considered negative. Then, differential expression analysis was also conducted on the 5 independent sets. IL7R, ITK, CD247 and CD3G were found to be relatively overexpressed in cluster A (FDR<0.05 and log2|FC|>1.0, **Figure S5B**). SORT1, GADD45A, PFKFB2 and IL18R1 were relatively expression-upregulated in cluster B (FDR<0.05 and log2|FC|>1.0, **Figure S5C**).

### Functional annotation of the two clusters

**Figure 4:**
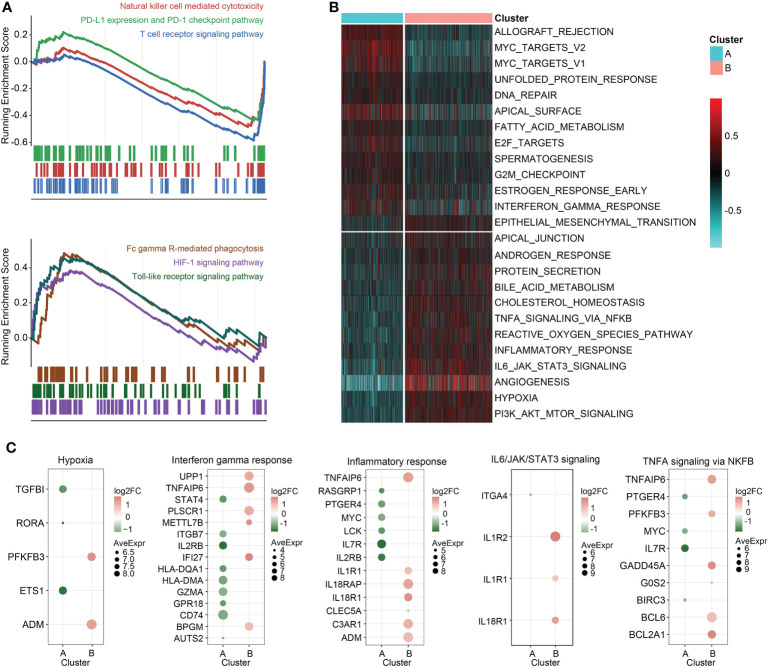
GSEA and GSVA. **(A)** GSEA of genesets for cluster A (top) and cluster B (bottom). **(B)** Heatmap of GSVA on sepsis samples grouped by K-means clusters. **(C)** Scatterplot of the changed pathway-related signatures.

Enrichment analysis on the overexpressed DEGs in cluster B revealed that NAD+ nucleosidase receptor, ADP-ribose, sugar-phosphatase kinase, and UDP-glucosyltransferase were significantly enriched (**Figure 3F**). Moreover, GO analysis of the overexpressed DEGs in cluster A demonstrated MHC antigen class II, cell non-membrane adaptor kinase, cytokine co-receptor growth activity, serine-type endopeptidase hydrolase peptidase, and scavenger receptor activity were remarkably enriched (**Figure 3G**). These findings were also detected in the independent validation datasets (**Table S6**). Then, the DEGs in the training set between the two clusters were ordered by the corresponding log2FC values. The GSEA was conducted using gseKEGG function in R package ‘clusterProfiler’, which demonstrated that cluster A was characterized by relatively upregulated immune activity, such as the upregulated natural killer cell mediated cytotoxicity, PD-L1 expression and PD-1 checkpoint pathway, and T cell receptor signaling pathway (**Figure 4A**, upper). In addition, Fc gamma R-mediated phagocytosis, and Toll-like receptor signaling pathway were significantly enriched in the cluster B (**Figure 4A**, lower). To validate the above findings, GSVA was carried out, with ‘h.all.v7.5.1.entrez.gmt’ as the reference. Biological processes such as interferon gamma response, and DNA repair were relatively in cluster A. And pathways such as TNFA signaling *via* NFκB, and IL6/JAK/STAT3 signaling were significantly enriched in cluster B (**Figure 4B**). Meanwhile, the log2FC values of the relative marker further validated the changed processes and pathways in GSVA step (**Figure 4C**).

### Immune infiltrates characteristics

**Figure 5:**
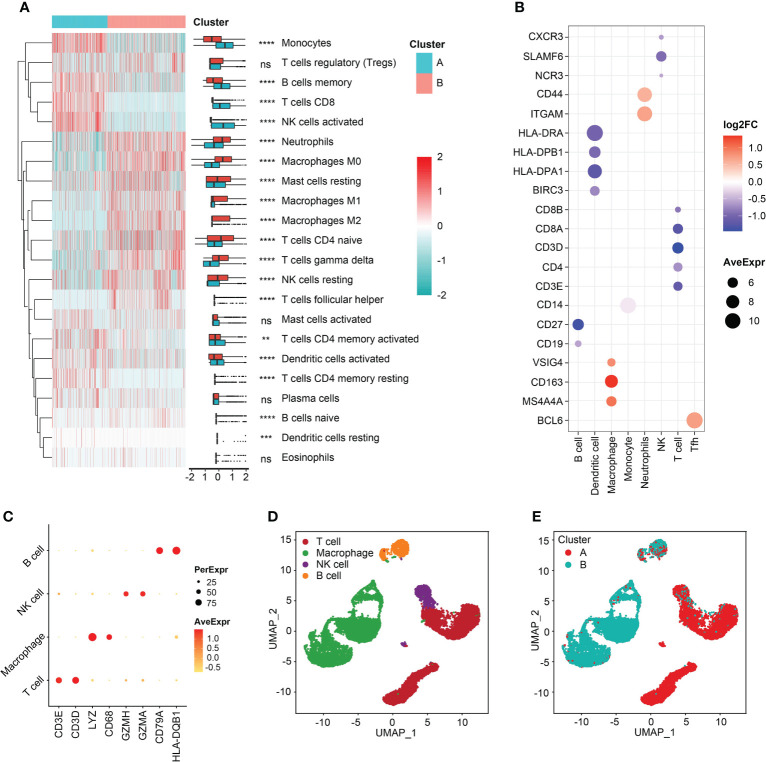
Immune reprogramming analysis. **(A)** Complex heatmap of immune cell fractions between cluster A and cluster B. **(B)** Scatter plot of log2FC values of immune cell markers. Color red represented the genes were relatively overexpressed in cluster B and color blue represented markers were comparatively upregulated in cluster A. **(C)** Scatter plot of markers expressed in single cell RNA-sequencing samples. **(D)** Cell annotation analysis identified four types of cells. **(E)** The distribution of four types of cells between the two clusters. **p < 0.01; ***p < 0.001; ****p < 0.0001; ns, not significant.

Immune cell-infiltrating patterns and signatures were systematically evaluated. After grouped by the clustered subtypes, we found CD8+ T cells, activated NK cells, memory B cells, monocytes, activated dendritic cells (aDCs), and activated T cells CD4 memory demonstrated increased infiltrating levels in cluster A (**Figure 5A**). In contrast, neutrophils, M0, M1, and M2 macrophages, naive CD4+ T cells, gamma delta (γδ) T cells, and resting NK cells were significant infiltrated in samples classified into cluster B (**Figure 5A**). Then, the immune-related signatures of T cells, DCs, macrophages, monocytes, neutrophils, NK cell, follicular helper T cells (Tfh) further validated that the infiltrating changes (**Figure 5B**). In the step of scRNA-seq analysis, we first used markers from CellMarker database to annotate the cells, and the markers were as follows: T cell (CD3E, and CD3D), macrophage (Lysozyme [LYZ] and CD68), NK cell (Granzyme A and H [GZMA and GZMH]), and B cell (CD79B and Major Histocompatibility Complex, Class II, DQ Beta 1 [HLA-DQB1]) (**Figure 5C**). The four cell clusters were displayed with the UMAP algorithm (**Figure 5D**). Then, we achieved two clusters based on the K-means clustering method, and significant differences of immune infiltrates could be observed. The cluster A was characterized by high infiltration of T cells and NK cells and the macrophages were specifically highly infiltrated in cluster B subgroup (**Figure 5E**). In addition, the divided two clusters had B cell infiltrations, to some degree (**Figure 5E**). In order to analyze the cytokine and chemokine milieu characterizing each cluster, we analyzed the expression of selected cytokine and chemokine mRNAs in the sepsis samples. Cluster B was associated with high expression of TGFβ pathway-relevant markers, a higher innate immune/decreased adaptive immune signal, which might indicate the cluster A could be defined as Inflammatory subtype. Expression of T lymphocytes-related mRNAs were relatively higher in cluster A and a reduced innate immune/higher adaptive immune signal, which suggested that this cluster may be classified as the Adaptive subphenotype (**Figure S6**).

### Feature selection

**Figure 6:**
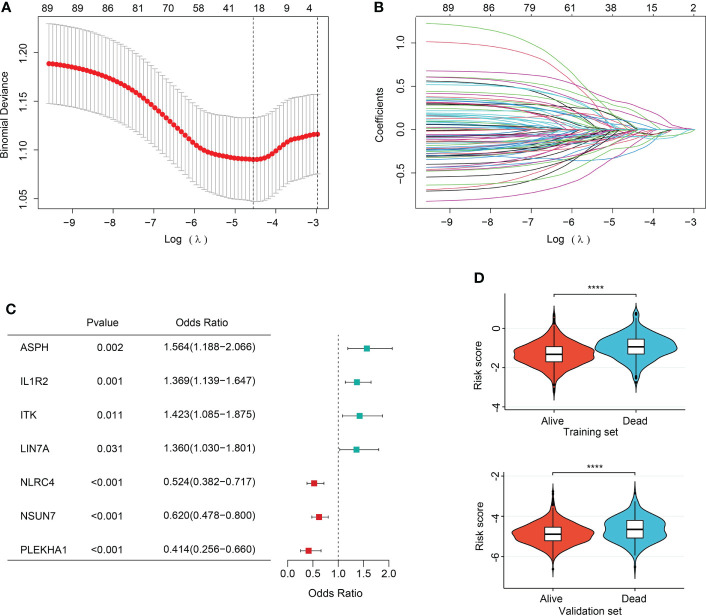
Feature selection and association of risk score with sepsis outcomes. **(A)** The ten-fold cross-validation results. The line on the left indicated the value of the parameter log(λ) for the error-minimized model. 28 variables were filtered out when log(λ) = −4.74. **(B)** LASSO coefficient profiles of the 28 features. **(C)** Forest plot of features significant in logistic regression analysis. **(D)** Violin plot of distribution of risk score between cluster A and cluster B in the training (upper) and validation (lower) sets.

In order to ever have any relevance to clinical outcomes, we need some way to determine cluster membership for any given new patient. Firstly, 90 sepsis-related DEGs were subjected to LASSO regression step with 10-fold cross validation. In the training set, following feature selection, 28 features were saved (**Figures 6A, B**). The filtered signatures were used to calculate risk score, including ASPH, ATP9A, CD247, CNIH4, DACH1, DOCK10, GADD45A, HK3, IL1R2, ITK, LIN7A, MAPK14, MGAM, MTR, NAIP, NLRC4, PEBP1, PLEKHA1, SAMD3, SIDT1, SIPA1L2, SLC7A6, SORT1, ST6GALNAC3, TXK, UBASH3A, UGCG, and NSUN7. Inclusion of these 28 variables in a logistic regression model resulted in 7 variables that were independently statistically significant predictors of clinical outcomes of sepsis patients (P<0.05, respectively) and were included in risk score. These variables included ASPH (OR, 1.564; 95% CI, 1.188-2.066; P=0.002), IL1R2 (OR, 1.369, 95% CI, 1.139-1.647, P=0.001), ITK (OR, 1.423, 95% CI, 1.085-1.875, P=0.011), LIN7A (OR, 1.360, 95% CI, 1.030-1.801, P=0.031), NLRC4 (OR, 0.524, 95% CI, 0.382-0.717, P<0.001), NSUN7 (OR, 0.620, 95% CI, 0.478-0.800, P<0.001), PLEKHA1 (OR, 0.414, 95% CI, 0.256-0.660, P<0.001) (**Figure 6C**).

### Risk score construction and performance evaluation

**Figure 7:**
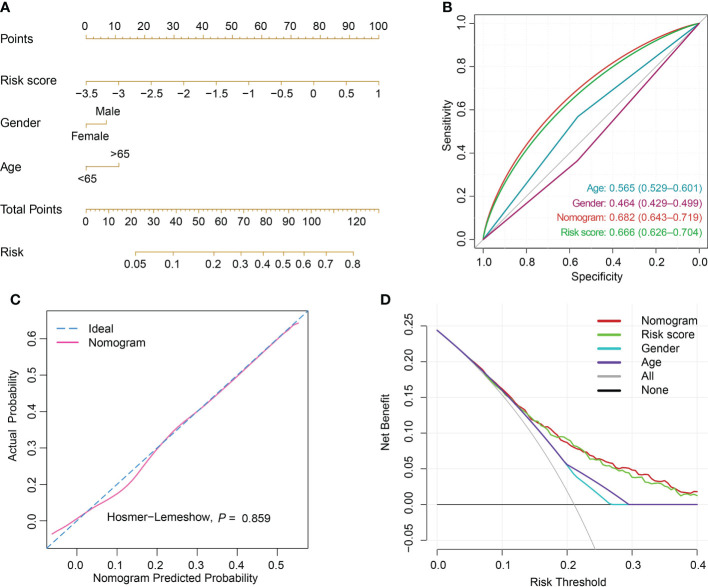
Nomogram establishment and performance assessment. **(A)** A nomogram established by multivariate logistic regression for predicting the risk of sepsis survival outcomes. **(B)** ROC curves demonstrated the capability of nomogram, risk score, age and gender in predicting prognosis of sepsis patients. **(C)** Calibration plot with a binary fringe plot of nomogram in the training set. **(D)** Decision curve analysis for the sepsis nomogram and age, gender and risk score.

The risk score was computed rooted on the coefficients from the logistic model and the corresponding expression values of the 7 candidates (**Table S7**). By comparison of the risk score values between alive and dead sepsis samples, there was a significant increase of the calculated score in non-survivor objects in training and validation sets (P<0.001, respectively, **Figure 6D**). These findings demonstrated the risk score might act as an indicator to predict the clinical outcomes of septic patients. Furthermore, by multivariate logistic regression analysis, the risk score was confirmed as an independent predictor for clinical outcomes of sepsis when adjusted by clinical characteristics such age and gender in training and validation sets (training set: OR, 2.704, 95% CI, 2.098-3.514, P<0.001; validation set: OR, 2.007, 95% CI, 1.469-2.759, P<0.001, **Table S8**). Then, the nomogram, including age, gender and risk score was constructed (**Figures 7A** and **S7A**). The ROC analysis demonstrated that the nomogram had great discriminative capacity than that computed based on risk score, or age, and or gender of sepsis patients (training set: AUC=0.682, 95% CI, 0.643-0.719; validation set: AUC=0.624, 95% CI, 0.576-0.664, **Figures 7B** and **S7B**). The risk scores calculated rooted on the 7 mRNAs demonstrated the great capacity in differentiating survivors from non-survivors with sepsis (training set: AUC=0.666, 95% CI, 0.626-0.704; validation set: AUC=0.608, 95% CI, 0.566-0.655). Calibration plots of the nomograms demonstrated that there were no untoward deviations of predicted risk from observed risk of sepsis outcomes over the entire range (**Figures 7C** and **S7C**). In DCA curves, the nomogram had a higher net benefit in terms of accurately detecting sepsis survival status, compared with that of age and gender and risk score (**Figures 7D** and **S7D**). The established sepsis response signatures (SRS) system which classify the sepsis patients into immunosuppressed, and immunocompetent subtypes and stratify clinical outcomes of sepsis patients (23). We tested the performance of such system in predicting sepsis prognosis and there was a relatively lower discriminative capacity in differentiating alive and dead sepsis patients (AUC=0.534, 95% CI, 0.451-0.617), in comparison with that of risk score in the training and validation sets. Additionally, in the training set, E-MTAB-4451, GSE65682 and GSE95233 included the survival information with a cutoff of 28d. Therefore, the three were treated as a whole for analysis. GSE185263 set defined survival status as whether in-hospital death occurred and uncertain cutoff values were introduced. Due to the differences in the time cut-off point of the four sets, we carried out ROC analysis for the specific cohort. The AUC demonstrated the risk score could predict the prognosis of sepsis (28d cutpoint: AUC: 0.681, 95%CI: 0.648-0.732; uncertain cutpoint: AUC: 0.616, 95%CI: 0.539-0.692, **Figure S8A**). In the validation set, E-MTAB-5273, E-MTAB-7581 and E-MTAB-4421 included the survival information with a cutoff of 28d. Similar to the above method. However, GSE95233 set had uncertain survival cutoff values. The AUC demonstrated the risk score could predict the prognosis of sepsis (28d cutpoint: AUC: 0.603, 95%CI: 0.550-0.657; uncertain cutpoint: AUC: 0.626, 95%CI: 0.504-0.727, **Figure S8B**).

### Interactions of risk score with clinical features and response to therapy

**Figure 8:**
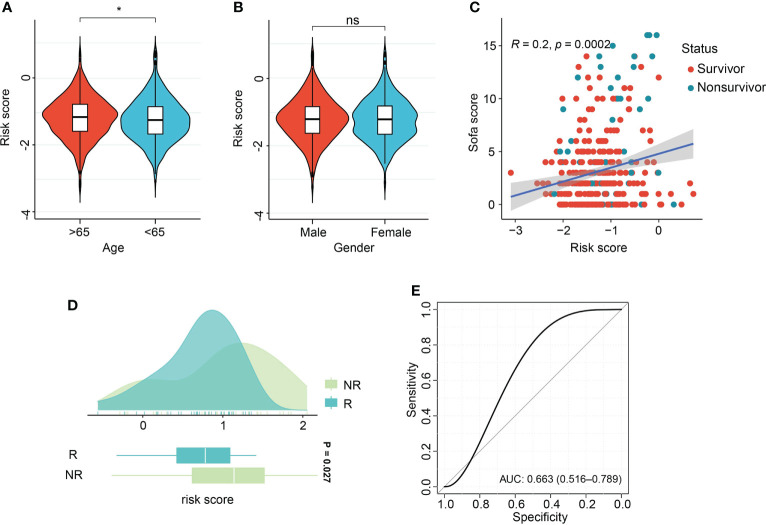
Association of risk score with clinical features and therapeutic response. **(A, B)** Violin plot of association of risk score with age and gender of sepsis patients. **(C)** Scatter plot of Pearson correlation analysis of risk score and sequential organ failure assessment (SOFA) score. **(D)** Box density plot of risk score with clinical therapeutic response. **(E)** ROC curve of performance of risk score in predicting early supportive therapy. *p < 0.05; ns, not significant.

Association of risk score with age and gender demonstrated the aged patients had comparatively increased risk score, in comparison with patients with age<65 years (**Figure 8A**). There were no differences of risk score between male and female patients (**Figure 8B**). Pearson correlation analysis demonstrated the positive relation of risk score and sequential organ failure assessment (SOFA) score (r=0.2, P=0.002, **Figure 8C**). SOFA score and acute physiology and chronic health evaluation (APACHE) II score are the most widely used and authoritative critical illness evaluation system. Therefore, the association of SOFA score, APACHE II score and risk score was calculated. By ROC analysis, we found SOFA score and APACHE II score performed better than risk score in the GSE185263 and GSE54514 sets (GSE185263: SOFA: AUC=0.699, 95%CI: 0.618-0.778; GSE54514: APACHE II: AUC=0.789, 95%CI: 0.706-0.856, **Figures S8C, D**). Given the risk score demonstrated great accuracy than age and gender of sepsis patients in predicting clinical outcomes, therefore, we integrated the risk score and clinicopathological features into a comprehensive model. After combining with risk score, age, gender, APACHE II score, the risk model outperformed the individual covariate (GSE185263: AUC=0.725, 95%CI: 0.631-0.793, **Figure S8C**). Similar findings were also obtained in the combination of risk score, age, gender, and SOFA score (GSE54514: AUC=0.823, 95%CI: 0.732-0.903, **Figure S8D**). The dataset GSE110487 includes the information related to the clinical response of septic shock patients to early supportive therapy (24). We examined whether there were interactions between risk score and the binary therapeutic responsive status. As shown in **Figure 8D**, increased risk scores were obtained in the patients responded to early supportive therapy (P=0.027). In addition, the ROC analysis was performed and revealed that risk score might be an effective tool to predict the response to clinical interventions (AUC=0.663, 95% CI, 0.516-0.789, **Figure 8E**).

## Discussion

Sepsis represents a variety of distinct disease states and displays in a number of different manners, such as fever, decreased vascular resistance (VR) and even multiple organ dysfunction and failure (25). Eventually, an imbalanced host response could lead to death in an individual who is suffering from sepsis, even with timely traditional interventions (26). Transcriptomic features that classify the host immune response will contribute to the development of novel therapeutic treatments the improvement of personalized management for sepsis (27). Prediction of clinical outcomes could be well accomplished by establishing the specific classifiers, which have been validated with transcriptomic data (28–30). Therefore, the purposes of the research were to reveal the clinical subtypes using large-scale samples with sepsis.

In the present study, K-means clustering analysis was carried out on transcriptomic profiles of sepsis (training set, n=1,692; validation set, n=617) from 12 sepsis datasets, revealing two robust sepsis subtypes. Previous research has confirmed the reliability of such machine-learning methods (27). The Inflammatory subphenotype was characterized by high expression of genes involved in pro-inflammatory (e.g., upregulation of inflammatory response) and innate immune reactions, demonstrating such type of sepsis might be involved in activation of innate immune response (31, 32). For example, overactivation of TNFA signaling *via* NFκB signaling, and IL6/JAK/STAT3 signaling has been identified to be associated with M1 macrophage polarization (31, 32). Upregulation of PI3K/AKT/MTOR signaling, and angiogenesis related to M2 macrophage polarization were also obtained in cluster B (33). A clear difference in cellular metabolism could be observed between the subclusters, for example, the increased activity of HIF signaling pathway in cluster B. Recent research demonstrates, hyperinflammatory status could increase glycolysis metabolism which elevates lactate production through activation of HIF signaling and promotes the production of proinflammatory molecules such as IL-1β and IL-6 (34, 35), which were consistent with our findings. In addition, compared to young patients, elderly patients undergo significant defects in humoral immune function (36), and declined expression of HLA-DR has been considered as a marker for on septic monocytes, resulting in the increase of clinical complications and poor outcomes (37), which might underlie the elderly patients with higher mortality rate in this cluster. In contrast, activation of adaptive immune response was relatively upregulated such as T cell receptor signaling pathway. Meanwhile, the samples in the Adaptive subcluster tended to be younger and demonstrated advantageous outcomes based on their clinical characteristics (38). Furthermore, pathways associated with both clusters suggested that these pathways were modulated in opposite directions, which further suggested by the strong inverse correlation between the subclusters in K-means and PCA analyses. The biological insights might contribute to the development of clinical treatment strategies for different subtypes. It has been shown that upregulation of innate immunity in early stages of sepsis is related to a higher mortality rate, however, the comparative absence of those changes and the expansion of adaptive immunity may have a positive effect on clinical outcomes (39). And our research further supported the previous findings. Uncovering sepsis heterogeneity might contribute to the improvement in development of therapies which might be beneficial to the specific subtype. There has been considerable attention paid to the role of the PD-1 pathway in the exhaustion of T cells and the suppression of anti-tumor immunity (40). In the field of severe infection, recent research reports that an increased percentage of PD-L1^+^ NK cells could support disease development and act as a hazardous factor for prognosis of sepsis patients (41). In addition, in sepsis-associated acute renal injury (ARI), the overexpressed PD-L1 in kidney could lead to immunosuppression due to the elevated level of lactate (42). Anti-PD-L1 therapeutic regimens have been tested in sepsis objects that are known to modulate the adaptive immune systems (43). A relevant study reported by Zhang et al. demonstrated that immune checkpoint blockade (ICB) could improve survival in experimental sepsis through inhibition of lymphocytic apoptosis and reversion of monocytic dysfunction (44). In the present research, immune checkpoint such as PD-L1 and relevant pathway were upregulated in the Adaptive cluster, which demonstrated that the ICB treatments might be more applicable to the Adaptive cluster. And the upregulation of PD-L1 expression and PD-1 checkpoint pathway might further explain the newly developed classification system for the application of anti-PD-L1 treatments, further research on which might illustrate the potential clinical utility.

It provides a basis for sophisticated methods and algorithms to better analyze high-dimensional data, especially these associated with clinicopathological characteristics, with the advancement and progression in multi-omics data (45). In disordered populations, subclusters could be explored and validated, based on the K-means clustering analysis. It has been observed that different patient endotypes are associated with different severity levels and varying mortality rates. Our research demonstrated a relation of the Inflammatory endotype with low adaptive immunity and high mortality in the training set, which was consistent with previous findings (46, 47). Previous studies on sepsis heterogeneity using clustering analysis have successfully demonstrated the subclasses of sepsis, and reveal the association of subphenotypes with clinical outcomes based on MARS, UK-based and US-based datasets (5, 12, 48). Although, the different outcomes among the for clusters have been identified by machine-learning, however, further quantification of cluster finding for sepsis patients and clinical application of the classification system has not been investigated. In our research, 7-gene survival model was computed using LASSO-logistic regression analysis in the discovery set (n=980) and validated in external datasets (n=760). The model displayed the prognostic value and positive correlations with SOFA score and aging, which was also identified as an independent predictor for clinical outcomes of sepsis patients (P<0.05, respectively). Additionally, Sepsis patients with low risk might benefit more from early supportive treatments, in comparison with the counterparts with low risk. Meanwhile, our risk model had better prognosis-predicting performance than the SRS classification system (SRS: AUC=0.534, 95% CI, 0.451-0.617; risk score in training set, AUC=0.666, 95% CI, 0.626-0.704; risk score in validation set: AUC=0.608, 95% CI, 0.566-0.655). In addition, the established nomogram included age, gender and risk score demonstrated higher prognosis-predicting performance than the individual covariate. These findings might provide evidence for clinical management of sepsis patients.

Our study has several limitations. Firstly, a wide range of public datasets associated with sepsis were included in the present research. Potential batch bias might be introduced, even with the help of algorithm in R package ‘sva’. Secondly, after merging the datasets, a great percentage of genes were not included, which might make several crucial molecules related to sepsis pathology lost during the processes. And even more, the missed key molecules might influence the accuracy and stability of K-means clustering findings. Thirdly, there were a small number of samples available in the public database used to investigate the association of risk score and treatment strategies in this study. An increase in sample size would elevate the statistical power of the predictive performance of risk model. Finally, in view of the incomplete information concerning other disorders and/or comorbidities in the included data sets, reproductivity of cluster findings and overall predictive performance of the risk model might not be confirmed with enough certainty. Further investigations are needed for validation of the prognostic model and K-means cluster analysis.

In conclusion, our study explored and validated two clusters of sepsis, which demonstrated distinctive mortality rate and response to early supportive therapy. Subcluster A was characterized by upregulation of innate immune response with disadvantageous clinical outcomes, whereas subphenotype B was demonstrated overactivation of adaptive immunity. In addition, a 7-gene risk model to predict sepsis survival was constructed, demonstrating great accuracy than SRS system. A nomogram was established for risk calculation in clinical practice.

## Data availability statement

The original contributions presented in the study are included in the article/**Supplementary Material**. Further inquiries can be directed to the corresponding author.

## Author contributions

CYZ and WZ analyzed and interpreted the patient data regarding the sepsis disease. ZWZ and CLZ performed the statistical analysis. ZWZ, JZ, CLZwere major contributors in writing the manuscript. All authors contributed to the article and approved the submitted version.

## Funding

This work was supported by the Medical Discipline Construction Project of Pudong Health Committee of Shanghai (PWYgy 2021-07) and the Outstanding Leaders Training Program of Pudong Health Bureau of Shanghai (PWR12018-07).

## Acknowledgments

We gratefully acknowledge Gene Expression Omnibus (GEO) and ArrayExpress databases which made the genomic data and clinical data of sepsis available.

## Conflict of interest

The authors declare that the research was conducted in the absence of any commercial or financial relationships that could be construed as a potential conflict of interest.

## Publisher’s note

All claims expressed in this article are solely those of the authors and do not necessarily represent those of their affiliated organizations, or those of the publisher, the editors and the reviewers. Any product that may be evaluated in this article, or claim that may be made by its manufacturer, is not guaranteed or endorsed by the publisher.
