# Supplementary material for: Identification of two robust subclasses of sepsis with both prognostic and therapeutic values based on machine learning analysis
Source: Front Immunol. 2022 Nov 25;13:1040286. doi: 10.3389/fimmu.2022.1040286 (PMC9732458; doi:10.3389/fimmu.2022.1040286)
Supplement: Supplementary file 1 [file DataSheet_1.docx]

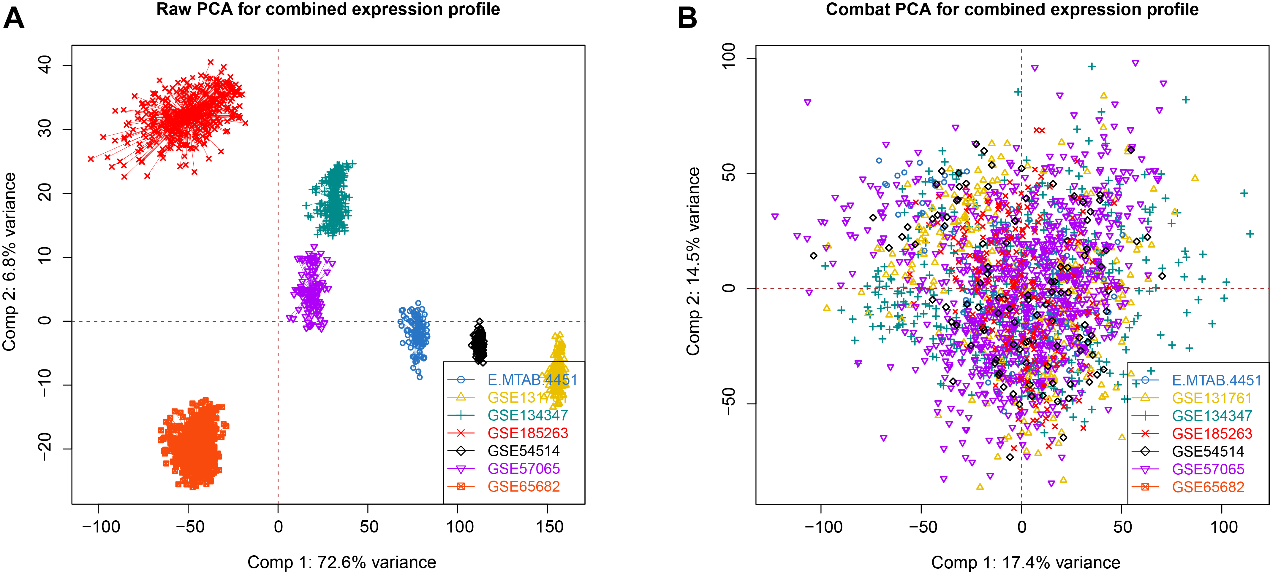


**Figure S1.** The clear batch effect among the 7 cohorts (A) and corrected by sva package (B).


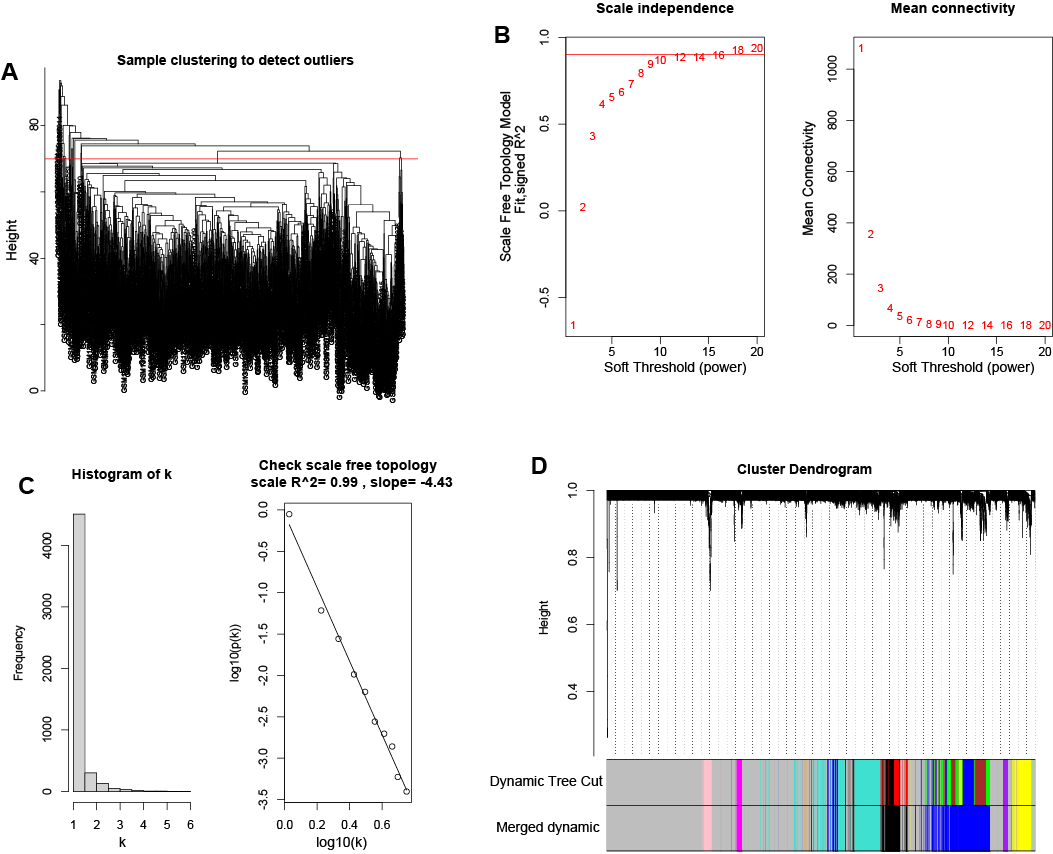


**Figure S2.** The sample clustering analysis for detecting outliers, and abline=70 was set (A). Soft power selection. The horizontal axis was the Soft threshold (power), and the vertical axis was the evaluation parameter of the scale-free network (B). The constructed network is more in line with the scale-free network characteristics (C). To merge the modules with similarity>0.8 (D).


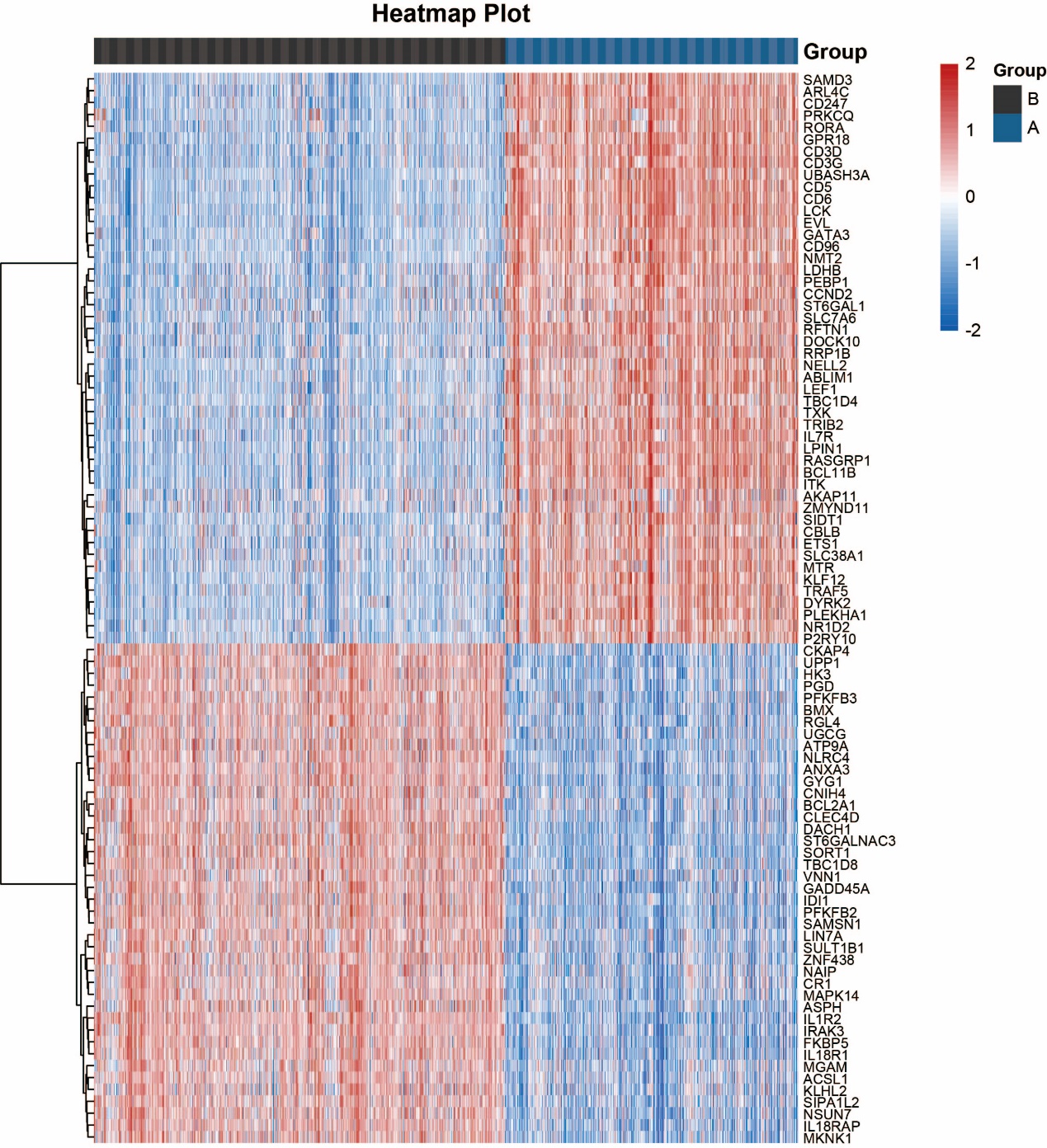


**Figure S3.** Heatmap demonstrated that distinct molecule features between cluster A and cluster B.


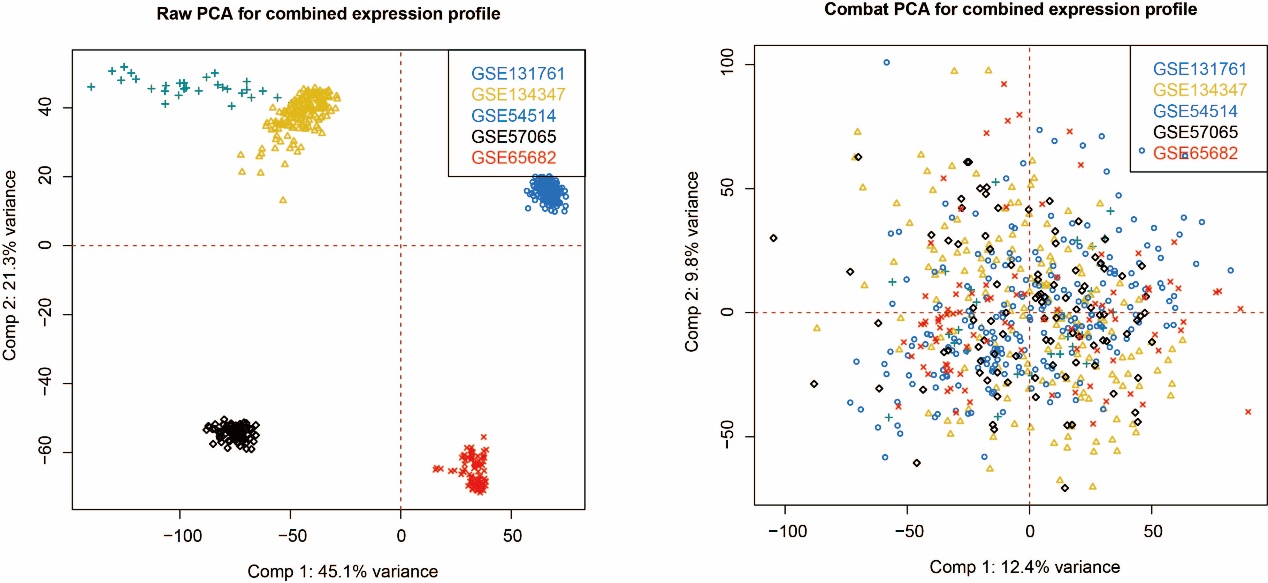


**Figure S4.** The clear batch effect among the 5 cohorts (A) and corrected by sva package (B).


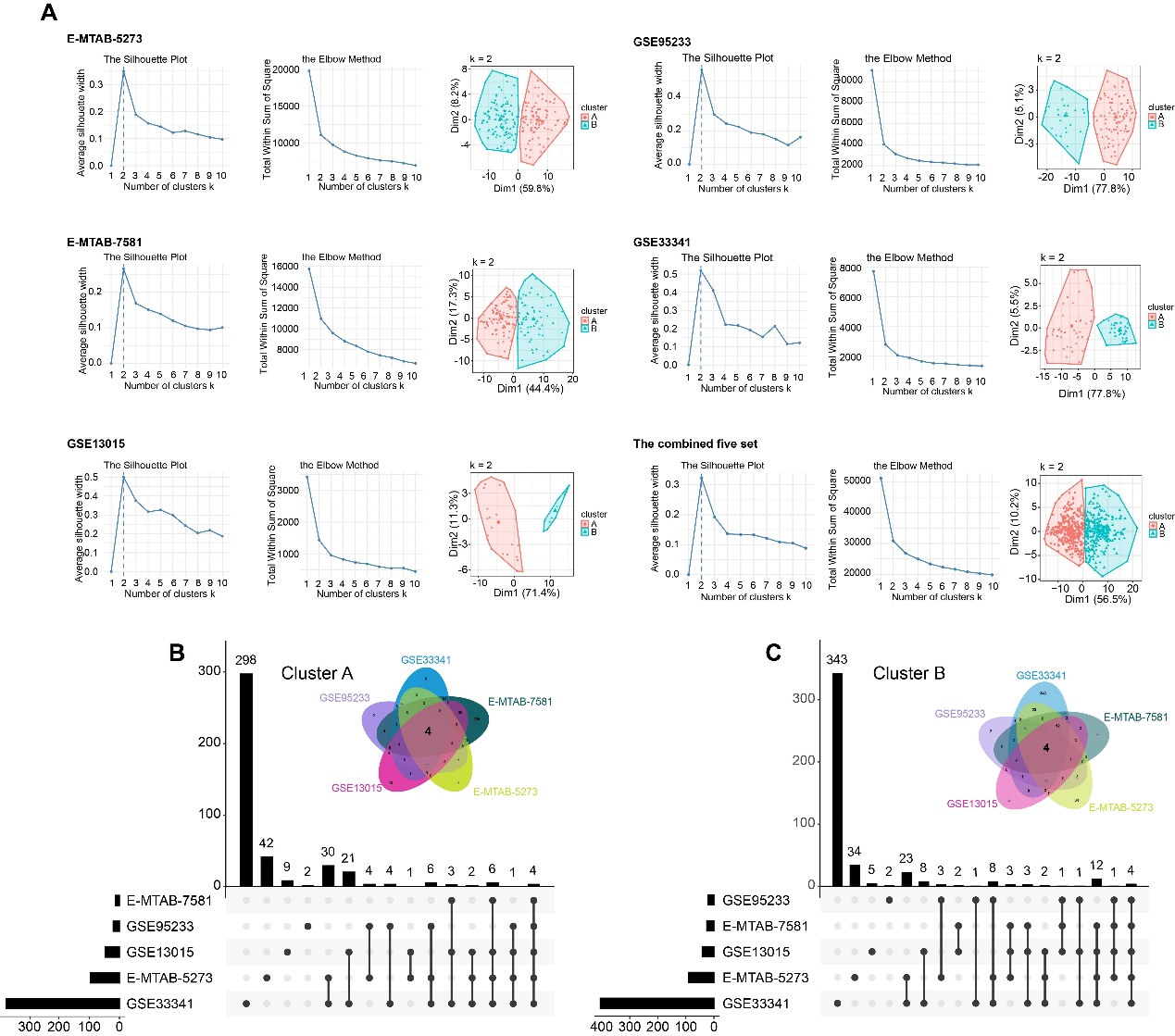


**Figure S5.** Validation of K-means clustering analysis in 5 independent cohorts (A). Upset plots and venn plots of differentially expressed genes between cluster A and cluster B in the 5 sets (B-C).


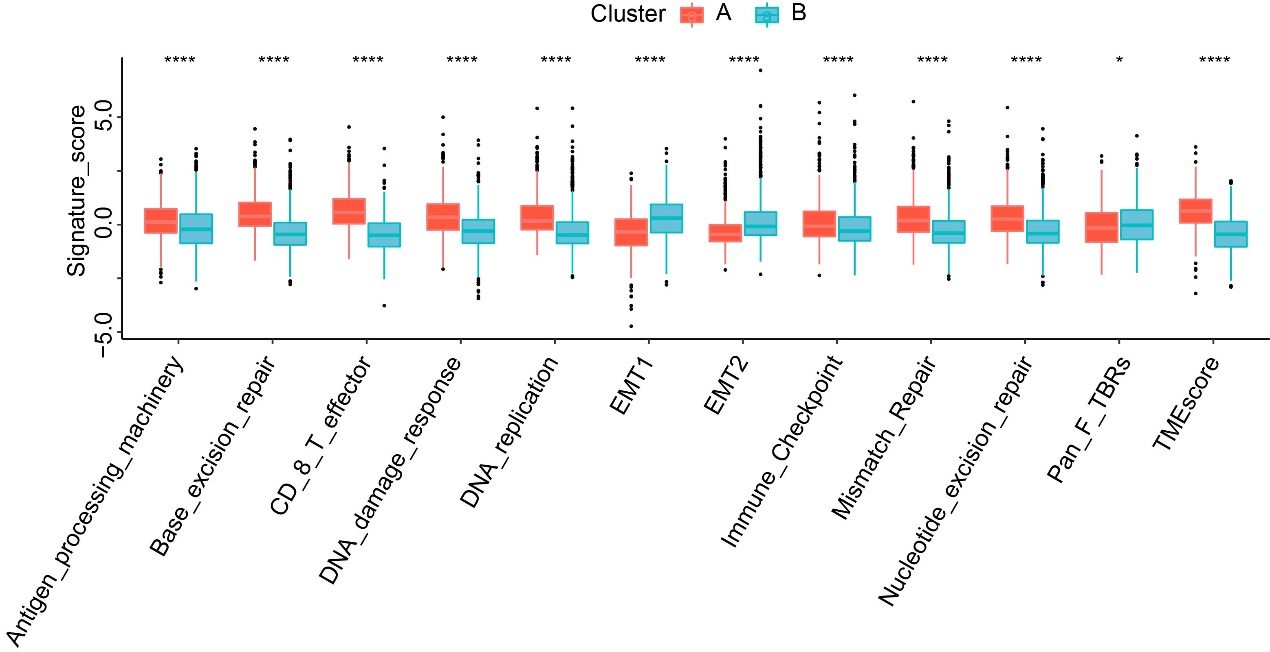


**Figure S6.** The boxplots of biological processes between clister A and cluster B.


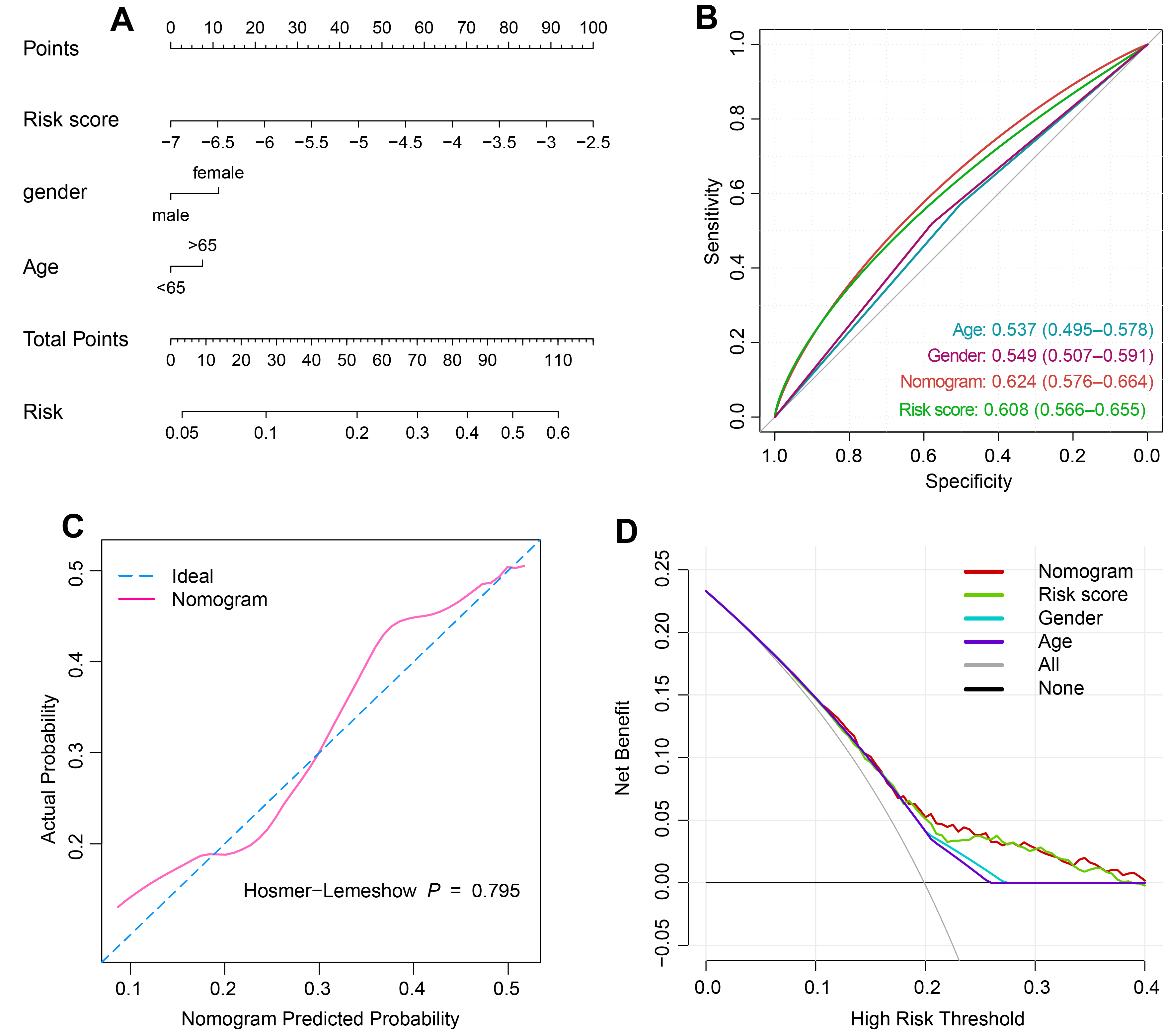


**Figure S7.** Nomogram establishment and performance assessment in the validation sets.

1. A nomogram established by multivariate logistic regression to predict the risk of sepsis survival outcomes.
2. ROC curves demonstrated the great accuracy of nomogram, risk score, age and gender in survival prediction.
3. Calibration plot with a binary fringe plot of nomogram in the validation set.
4. Decision curve analysis for the sepsis nomogram and age, gender and risk score in the validation set.


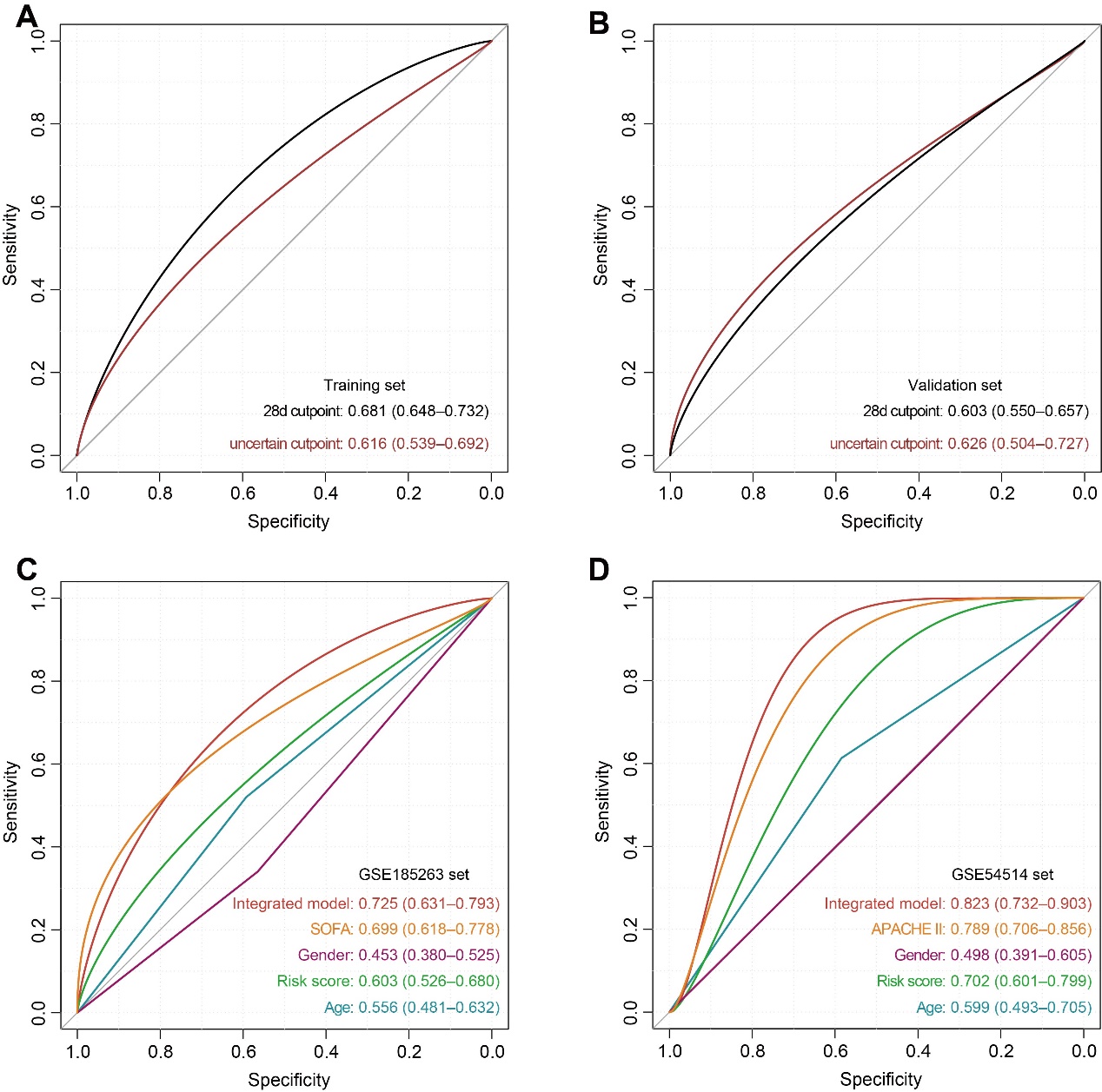


**Figure S8.** The AUC demonstrated the risk score could predict the prognosis of sepsis **a**t different time cutpoint in the training (A) and validation (B) sets. The integrated model including risk score and clinicopathological features revealed great accuracy than the individual risk factors in GSE185263 (C) and GSE54514 (B) sets.
